# Non-negative discriminative brain functional connectivity for identifying schizophrenia on resting-state fMRI

**DOI:** 10.1186/s12938-018-0464-x

**Published:** 2018-03-13

**Authors:** Qi Zhu, Jiashuang Huang, Xijia Xu

**Affiliations:** 1 College of Computer Science and Technology, Nanjing University of Aeronautics and Astronautics, Nanjing, 210016 China; 2 Collaborative Innovation Center of Novel Software Technology and Industrialization, Nanjing, 210093 China; 3 Department of Psychiatry, Affiliated Nanjing Brain Hospital, Nanjing Medical University, Nanjing, 210029 China

**Keywords:** Schizophrenia, Computer aided diagnosis, Functional connectivity, Feature selection, Schizophrenia classification, Resting-state fMRI

## Abstract

**Background:**

Schizophrenia is a clinical syndrome, and its causes have not been well determined. The objective of this study was to investigate the alteration of brain functional connectivity between schizophrenia and healthy control, and present a practical solution for accurately identifying schizophrenia at single-subject level.

**Methods:**

24 schizophrenia patients and 21 matched healthy subjects were recruited to undergo the resting-state functional magnetic resonance imaging (rs-fMRI) scanning. First, we constructed the brain network by calculating the Pearson correlation coefficient between each pair of the brain regions. Then, this study proposed a novel non-negative discriminant functional connectivity selection method, i.e. non-negative elastic-net based method (N2EN), to extract the alteration of brain functional connectivity between schizophrenia and healthy control. It ranks the significance of the connectivity with a uniform criterion by introducing the non-negative constraint. Finally, kernel discriminant analysis (KDA) is exploited to classify the subjects with the selected discriminant brain connectivity features.

**Results:**

The proposed method is applied into schizophrenia classification, and achieves the sensitivity, specificity and accuracy of 100, 90.48 and 95.56%, respectively. Our findings also indicate the alteration of functional network can be used as the biomarks for guiding the schizophrenia diagnosis. The regions of cuneus, superior frontal gyrus, medial, paracentral lobule, calcarine fissure, surrounding cortex, etc. are highly relevant to schizophrenia.

**Conclusions:**

This study provides a method for accurately identifying schizophrenia, which outperforms several state-of-the-art methods, including conventional brain network classification, multi-threshold brain network based classification, frequent sub-graph based brain network classification and support vector machine. Our investigation suggested that the selected discriminant resting-state functional connectivities are meaningful features for classifying schizophrenia and healthy control.

## Background

Schizophrenia is a severely debilitating psychiatric disease, and its symptoms include obstacles in thinking, perception, emotion, behavior, etc. [1]. According to estimation of the world health organization, the global lifetime risk of schizophrenia is about 3.8–8.4‰. Schizophrenia poses a huge burden on patients, families and society. So it is very important to investigate the accurate diagnosis method for schizophrenia.

Schizophrenia is often considered to be a disorder of connectivity between brain regions. However, among the large-scale brain network, the alteration of the connectivity between schizophrenia and healthy control has not been well investigated. With advance in machine learning [2, 3] and modern imaging technologies, e.g., functional magnetic resonance imaging (fMRI) providing the functional interaction of the brain regions, more and more attention has been focused on machine learning based disease diagnosis using fMRI [4–8].

In general, the machine learning based diagnosis methods using fMRI can be summarized as two categories, i.e. the voxel based methods and the brain network [9–11] based methods. The voxel based methods need to construct the correlation model for each pair of voxels. For example, Demirci et al. used independent component analysis (ICA) to obtain independent component (IC) spatial maps from the voxels of fMRI data, and exploited projection pursuit algorithm for classification [8]. A three-phase feature selection method was proposed for diagnosis of schizophrenia using fMRI [12]. Cao et al. exploited sparse representation classification for voxel based schizophrenia diagnosis and biomarker selection [13]. It is usually hard to obtain enough schizophrenia participants to construct robust voxel network model, and the high computational cost also constrains its application. Besides above voxel based methods, brain network based methods also have been applied into neurodegenerative diseases diagnosis including schizophrenia and Alzheimer’s disease [14]. Most of these methods first constructed the brain functional network by measuring the correlation between each pair of brain regions, and then they selected the significant regions with different metrics, e.g. clustering coefficient [14] and Bayesian information criterion (BIC) [15]. Compared to the voxel based methods, the brain network based methods has higher classification efficiency and can provide more intuitive biomarkers for diagnosis.

The mental activity occurring during rest is relevant to the phenomenology of schizophrenia [16]. The resting-state functional network analysis is potentially useful in revealing the pathophysiology of schizophrenia. Compared to task-driven analysis, resting-state functional analysis can provide more complete and accurate brain network features for identifying schizophrenia [17]. Many machine learning algorithms were developed to extract discriminative features from resting-state functional network for schizophrenia [18–20]. Shen et al. [21] proposed to use a correlation coefficient method to select the highly discriminative functional connectivity features from resting-state functional network, and then they mapped these features into low-dimensional space by using locally linear embedding (LLE). They found the data in mapping space can reveal the distinct differences of functional patterns between schizophrenia and healthy control. Lynall et al. [22] investigated the some topological properties of the functional connectivity network, and found schizophrenia tends to have more diverse profile of brain functional network. Castro et al. [23] proposed to use multiple kernel learning to fuse the magnitude and phase data in fMRI, and achieved promising result in schizophrenia classification. By using multivariate statistics, Pergola et al. [24] found accounting for the alternation of thalamic would improve the detection of subjects at familial risk for schizophrenia.

In most of the schizophrenia classification problems, the size of the subjects is limited and the number of the features, such as brain connectivities and voxels, is very large. Schizophrenia classification with fMRI data is a typical small sample size learning problem, which leads curse of dimensionality, overfitting and poor generalization ability for classification algorithms. It is well known that feature selection stage plays an important role in improving the classification performance. Therefore, it is very necessary to design an effective feature selection method for schizophrenia classification problem.

Lasso [25, 26] is one of the most famous feature extraction methods. It aims to identify the most significant features for a compact data representation by constructing $l_{1}$-norm constraint based sparse representation model between feature and label. In Lasso, most of the sparse representation coefficients are equal or close to zero, and sparse representation coefficients with large amplitude are preserved, which makes it easy to explain the significances of the features in nature. Lasso and its variants have been widely used for removing redundant features in brain disease diagnosis. Rashid et al. exploited Lasso for estimating the dynamic connectivity from resting fMRI and identify differences among schizophrenia and other neurodegenerative diseases [14]. Zhang et al. introduced the group structure into Lasso and applied it into identifying Alzheimer’s disease and its early stage. Fused Lasso exploits the ordering of data by explicitly regularizing the differences between neighboring samples [27]. Watanabe et al. used the fused Lasso regularized support vector machine to account for the high dimensional correlation maps of brain [28].

Suppose we have p subjects and each subject has m brain connectivity or voxel features (p ≪ m). Lasso can select at most p features from the data because of the convex optimization problem. On one hand, the limited features selected by Lasso are often difficult to descript the difference between healthy control and schizophrenia in complex brain structure and function. On the other hand, if there are some pairwise correlations among a group of connectivity or voxel features, Lasso selects only one feature and does not consider which one to select. By combining $l_{1}$-norm and $l_{2}$-norm regularizations in regression model, Zou et al. proposed elastic-net feature selection algorithm [29]. Elastic-net takes both automatic feature selection and continuous shrinkage into account, and it can select groups of correlated variables. In elastic-net, the weights of two regular terms can be dynamically adjusted, which provides a flexible way to extract the discriminative connectivities for identifying schizophrenia. In addition, the conventional feature extraction methods often cannot rank the significances of the brain connectivities with a uniform criterion. For example, in Lasso, the significance value of each connectivity could be positive or negative. Both the sign and absolute value of the significance have effect on the rank of the connectivities.

**Fig. 1:**
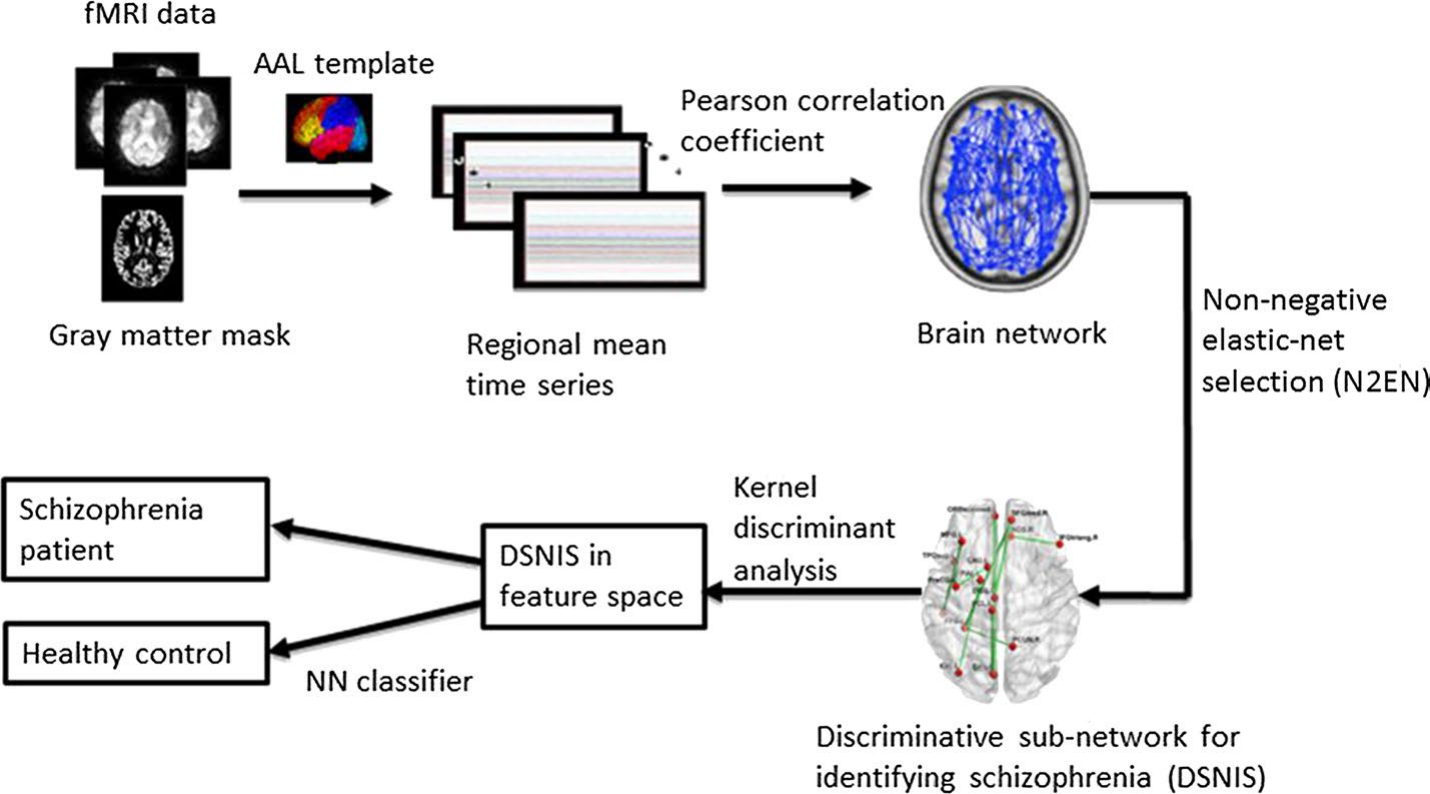
Flowchart of the proposed method

In this paper, we propose a novel feature extraction method, i.e. non-negative elastic-net based method (N2EN), for robust classification of healthy schizophrenia patients and healthy controls. Figure 1 shows the flowchart of the proposed method. We first use the N2EN to select the most significant connectivities from the whole brain network, and obtain the discriminative sub-network for schizophrenia. The N2EN is a multivariate representation model based on the connectivities, which can reflect the global structure of brain network. In N2EN, the significances of the brain connectivities are all positive, so we can rank the brain connectivities according to the absolute values of the significances. Then, we project the features of discriminative sub-network into reproducing kernel Hilbert space by kernel discriminant analysis (KDA) [30], in which the projection is obtained by maximizing the between-class covariance and minimizing the within-class covariance. Finally, the nearest neighbor classifier is used for classification. We applied the proposed method into schizophrenia identification. The results show that our method outperforms conventional brain network based methods and voxel based methods in identifying schizophrenia.

## Methods

### Participants

In this study, all participants were recruited from the department of psychiatry, affiliated Nanjing brain hospital of Nanjing medical university. 24 schizophrenia patients were recruited. All the recruited subjects were diagnosed by expert consensus panels. We used the positive and negative syndrome scale (PANSS) to obtain the score of severity of schizophrenia. PANSS is a widely used medical scale for measuring symptom severity of schizophrenia patients [31]. For each patient, an approximately 45-min clinical interview is conducted. The patient is rated from 1 to 7 on 30 different symptoms based on the interview as well as reports of family members or primary care hospital workers. The higher the PANSS score is, the more serious the symptoms are. Patients were not included if they had a prior history of organic brain diseases, infectious diseases, alcohol or drug abuse. 21 healthy subjects with matched age, gender, and average education were recruited as a control group. The inclusion criteria are as follows: (1) No mental disorder, and the current state of mind was in good condition; (2) Without a family history of psychosis in the three generations. (3) Aged from 18 to 50 years old. (4) Without organic brain disease, infectious diseases or other chronic somatic diseases. Table 1 shows demographic characteristics and clinical variables of the participants.

**Table 1:** Demographic characteristics and clinical variables of the participants

Diagnosis	Number	Age	Gender (F/M)	PANSS
Patients	24	30.78 ± 9.01	14/10	97.83 ± 11.09
Healthy controls	21	35.29 ± 7.94	15/6	Null

### Image acquisition

Data acquisition was performed using a 3-T Siemens Tim-Trio scanner with a 12-channel head coil. The resting-state fMRI (R-fMRI) images of each participant were acquired with the following parameters: flip angle = 90°, TR/TE = 2000/30 ms, imaging matrix = 64 × 64, FOV = 256 × 256 mm, 36 slices, 180 volumes, and voxel thickness = 3 mm. During scanning, all subjects were instructed to keep their eyes open and stare at a fixation cross in the middle of the screen for 5 min.

### Pre-processing

For each participant, the image prepossessing step is as follows: the first 10 volumes of functional time series were discarded because of the instability of MRI signal. Then, the leaving volumes were slice acquisition corrected, head-motion corrected, normalized to the SPM5 Montreal Neurological Institute template, and re-sampled to 3-mm^3^ voxels. After linear detrending, data was filtered using typical temporal bandpass (0.01–0.08 Hz), slow-5 bandpass (0.01–0.027 Hz), and slow-4 bandpass (0.027–0.073 Hz), respectively. Next, the motion parameters, the global mean signal, WM, CSF as nuisance covariates were used to reduce the effects of head motion and non-neuronal BOLD fluctuations.

### Identifying the alteration of connectivity by N2NE

**Fig. 2:**
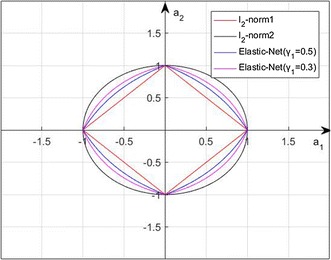
The comparison of different norms ($\gamma_{2}^{{}} = 1 - \gamma_{1}^{{}}$)

Suppose we have the time series from *m* brain regions with a system of fMRI volumes. We can construct the brain functional network by the commonly used Pearson correlation coefficient [2]. Among a large number of brain connectivities, it is necessary to extract the alteration or discriminative connectivities for schizophrenia. Let *x*_*i, j*_ denote the *i*th connectivity feature of the brain network from *j*th subject, and $y_{j}$ denotes the class label of *j*th subject. For estimating the significance of the connectivity, we construct the following model:

1 $$y = Xa + e$$

where $X = \{ x_{i,j} \} {\kern 1pt}$ $(i = 1,2, \ldots ,m,{\kern 1pt} {\kern 1pt} {\kern 1pt} j = 1,2, \ldots ,n)$, $y = [y_{1} ,y_{2} , \ldots ,y_{n} ]^{T}$, $a = [a_{1} ,a_{2} , \ldots ,a_{m} ]^{T}$, and *e* denotes the model error. Using $l_{2}$-norm or Tikhonov regularization to prevent over-fitting, the vector of regression coefficients can be obtained by solving the following problem:

2 $$\hbox{min}\,||y - Xa||_{2}^{2} + \gamma\, ||a||{\kern 1pt}_{ 2}^{ 2}$$

where $\gamma$ is the regular parameter. The coefficient vector $a$ obtained by above model is not sparse, which implies it is difficult to select the discriminative feature by comparing the values of these coefficients. Furthermore, there are positive and negative values in representation coefficients, which lead to the significance of the connectivity hard to rank with a uniform criterion. In this paper, we proposed non-negative sparse representation and ridge regression to guarantee an optimum. The objective function is as follows:

3 $$\hbox{min}\,||y - Xa||_{2}^{2} + \gamma_{1}^{{}} ||a||{\kern 1pt}_{1}^{{}} {\kern 1pt} + \gamma_{2}^{{}} ||a||{\kern 1pt}_{2}^{{}} {\kern 1pt} {\kern 1pt} {\kern 1pt} {\kern 1pt} {\kern 1pt} {\kern 1pt} {\kern 1pt} {\kern 1pt} s.t.{\kern 1pt} {\kern 1pt} {\kern 1pt} {\kern 1pt} \forall i:a_{i} > 0$$

where $||.||{\kern 1pt}_{1}^{{}}$ denotes the $l_{1}$-norm of the vector, e.g. $||a||{\kern 1pt}_{1}^{{}} = \sum {|a_{i} } |$. $\gamma_{1}^{{}}$ and $\gamma_{2}^{{}}$ are the non-negative parameter controlling the combination of the two regular terms. Figure 2 shows the comparison between the mixed norms used in elastic-net and the other norms. We define the following two augmented sets:

4 $$X{\kern 1pt}^{*} = (1 + \gamma_{2} )^{ - 1/2} \left( \begin{aligned} X \hfill \\ \sqrt {\gamma_{2} } I \hfill \\ \end{aligned} \right),\quad y{\kern 1pt}^{*} = \left( \begin{aligned} y \hfill \\ 0 \hfill \\ \end{aligned} \right)$$

The size of $X^{*}$ and $y{\kern 1pt}^{*}$ are $(n + m) \times m$ and $n + m$, respectively. Let $\alpha = \gamma_{1} (1 + \gamma_{2} )^{ - 1/2}$ and $a^{*} = (1 + \gamma_{2} )^{ - 1/2} a$. Then the objective function in Eq. (3) becomes

5 $$\hbox{min}\,||y^{*} - X^{*} a^{*} ||_{2}^{2} + \alpha ||a^{*} ||{\kern 1pt}_{1} {\kern 1pt} {\kern 1pt} {\kern 1pt} {\kern 1pt} {\kern 1pt} {\kern 1pt} s.t.{\kern 1pt} {\kern 1pt} {\kern 1pt} {\kern 1pt} \forall i:a_{i}^{*} > 0$$

So computing the solution of the proposed feature selection problem is equivalent to solving a non-negative sparse representation problem on augmented sets.

Let $b = (\alpha /2)v - X^{*T} y^{*}$, where $v$ is the vector whose elements are all 1, Eq. (5) is equivalent to:

6 $$\hbox{min}\,a^{*T} X^{T} Xa^{*} + 2b^{T} X{\kern 1pt} {\kern 1pt} {\kern 1pt} {\kern 1pt} {\kern 1pt} {\kern 1pt} s.t.{\kern 1pt} {\kern 1pt} {\kern 1pt} {\kern 1pt} \forall i:a_{i}^{*} > 0$$

Because $X^{T} X$ is nonnegative, symmetrical and semi-definite, the above problem is convex and has globally optimal solution. The non-negative elastic-net feature selection not only provides interpretation to the importance of the connectivity between two brain regions, but also tolerates data noise existing in brain network to some extent. We perform the non-negative elastic-net on brain network data, and the selected connectivities form a brain sub-network, which is referred to as discriminative sub-network for identifying schizophrenia (DSNIS). The ratio of the number of removed connectivities and the number of all the connectivities in original brain network are defined as the sparsity of DSNIS. The DSNIS can be expected has strong ability to simultaneously identify schizophrenia and evaluate the importance of the connectivity between two brain regions for schizophrenia.

Elastic-net is useful when multiple features are associated with another feature. Because, in above case, Lasso tends to pick one of them at random, while the elastic-net prefers to pick two, which alleviates the small sample size problem in schizophrenia classification. The non-negative elastic-net is a unified model to non-negative Lasso and ridge regression. When $\gamma_{1}^{{}} = 0$, the non-negative elastic-net degrades into non-negative ridge regression. When and $\gamma_{2}^{{}} = 0$, it degrades into non-negative Lasso. The proposed non-negative elastic-net is a well-defined convex optimization problem, which ensures the existence and uniqueness of solution.

### Classification with significant alteration connectivity

Considering most of the brain network data are linearly non-separable, for achieving good classification performance, we exploit KDA, i.e. a nonlinear method, for classification. Suppose we have a set of DSNIS samples, let $Z = \{ z^{i} \} ,\;i = 1,2, \ldots ,n$. By the nonlinear mapping $\phi$ induced by the kernel function *f*, each sample is transformed into the feature space and becomes $\phi (z^{i} ),c = 1,2, \ldots ,n$. In the feature space, KDA seeks the projection directions on which the samples from different classes are far from each other and samples from same class are close to each other simultaneously. The objective function of KDA is as follows:

7 $$\begin{aligned} {\kern 1pt} w_{opt} & = \arg \hbox{max} \frac{{w^{T} S_{b}^{\phi } w}}{{w^{T} S_{w}^{\phi } w}} \\ S_{b}^{\phi } & = \sum\limits_{i = 1}^{c} {n_{i} (u_{\phi }^{i} - u_{\phi } )(u_{\phi }^{i} - u_{\phi } )^{T} ,} {\kern 1pt} {\kern 1pt} {\kern 1pt} {\kern 1pt} {\kern 1pt} S_{w}^{\phi } = \sum\limits_{j = 1}^{c} {\sum\limits_{i = 1}^{{n_{c} }} {(\phi (z_{i}^{j} ) - u_{\phi }^{i} )(\phi (z_{i}^{j} ) - u_{\phi }^{i} )^{T} } } \\ \end{aligned}$$

where *c* is the class number, $n_{i}$ is the sample number of *i*th class, and $z_{i}^{j}$ represents the *i*th sample from *j*th class; $u_{\phi }^{{}}$ and $u_{\phi }^{i}$ are the global centroid and the centroid of *i*th class in the feature space, respectively; $S_{b}^{\phi }$ and $S_{w}^{\phi }$ are the between-class scatter matrix and within-class scatter matrix in the feature space, respectively. We also define the total scatter matrix in feature space.

8 $$S_{t}^{\phi } = \sum\limits_{j = 1}^{c} {\sum\limits_{i = 1}^{{n_{c} }} {(\phi (z_{i}^{j} ) - u_{\phi }^{i} )(\phi (z_{i}^{j} ) - u_{\phi }^{i} )^{T} } }$$

Because $S_{t}^{\phi } = S_{b}^{\phi } + S_{w}^{\phi }$, the Eq. (7) is equivalent to:

9 $$w_{opt} = \arg \hbox{max} \frac{{w^{T} S_{b}^{\phi } w}}{{w^{T} S_{t}^{\phi } w}}$$

which can be solved by the following Eigen-problem:

10 $$S_{b}^{\phi } w = \lambda S_{w}^{\phi } w$$

According to reproducing kernel theory, the projection *w* can be represented as:

11 $$w = \sum\limits_{i = 1}^{n} {\alpha_{i} \phi (z_{i} )}$$

where $\alpha = [\alpha_{1} ,\alpha_{2} , \ldots ,\alpha_{n} ]$. Substituting Eq. (11) into Eq. (9), and then we have

12 $$\alpha_{opt} = \arg \hbox{max} \frac{{\alpha^{T} KWK\alpha }}{{\alpha^{T} KK\alpha }}$$

where *K* is defined as $K_{i,j} = \phi (z_{i} )^{T} \phi (z_{j} ) = f(z_{i} ,z_{j} )$, and *W* is defined as:

13 $$W_{i,j} = \left\{ \begin{aligned} & 1/n_{c} ,\quad {\kern 1pt} {\kern 1pt} if{\kern 1pt} {\kern 1pt} z_{i} {\kern 1pt} {\kern 1pt} and{\kern 1pt} {\kern 1pt} z_{j} {\kern 1pt} {\kern 1pt} are{\kern 1pt} {\kern 1pt} from{\kern 1pt} {\kern 1pt} {\kern 1pt} the{\kern 1pt} {\kern 1pt} same{\kern 1pt} {\kern 1pt} class{\kern 1pt} \hfill \\ & 0,{\kern 1pt} \quad otherwise \hfill \\ \end{aligned} \right.$$

If *KK* is singular, we perform Eigen-decomposition on *K* for obtaining a stable solution of the problem in Eq. (12). For a sample *z*, its projection in feature space along direction *w* is:

14 $$\phi (z)^{T} w = \phi (z)^{T} \sum\limits_{i = 1}^{n} {\alpha_{i} \phi (z_{i} )} = \alpha K(:,i)$$

where $K(:,i)$ is the ith column of *K*. Finally, nearest neighbor classifier is used for disease classification.

## Results

### Classification for schizophrenia

We applied the proposed method to schizophrenia classification, and compared our method with conventional brain network classification (CNC) [32], multi-threshold brain network based classification (MTNC) [33], frequent sub-graph based brain network classification (FSGNC) [34] and support machine vector (SVM) [20]. Accuracy, sensitivity, specificity, positive predictive value (PPV), and negative predictive value (NPV) are measured to evaluate the performance of these methods, and they can be calculated as follows.

15 $$accuracy = \frac{TP + TN}{TP + FN + TN + FP}$$

16 $$sensitivity = \frac{TP}{TP + FN}$$

17 $$specificity = \frac{TN}{TN + FP}$$

18 $$PPV = \frac{TP}{TP + FP}$$

19 $$NPV = \frac{TN}{FN + TN}$$

where $TP$, $TN$, $FP$ and $FN$ are the number of the patients correctly predicted, healthy controls correctly predicted, healthy controls predicted as patients, and patients predicted as healthy controls, respectively.

**Fig. 3:**
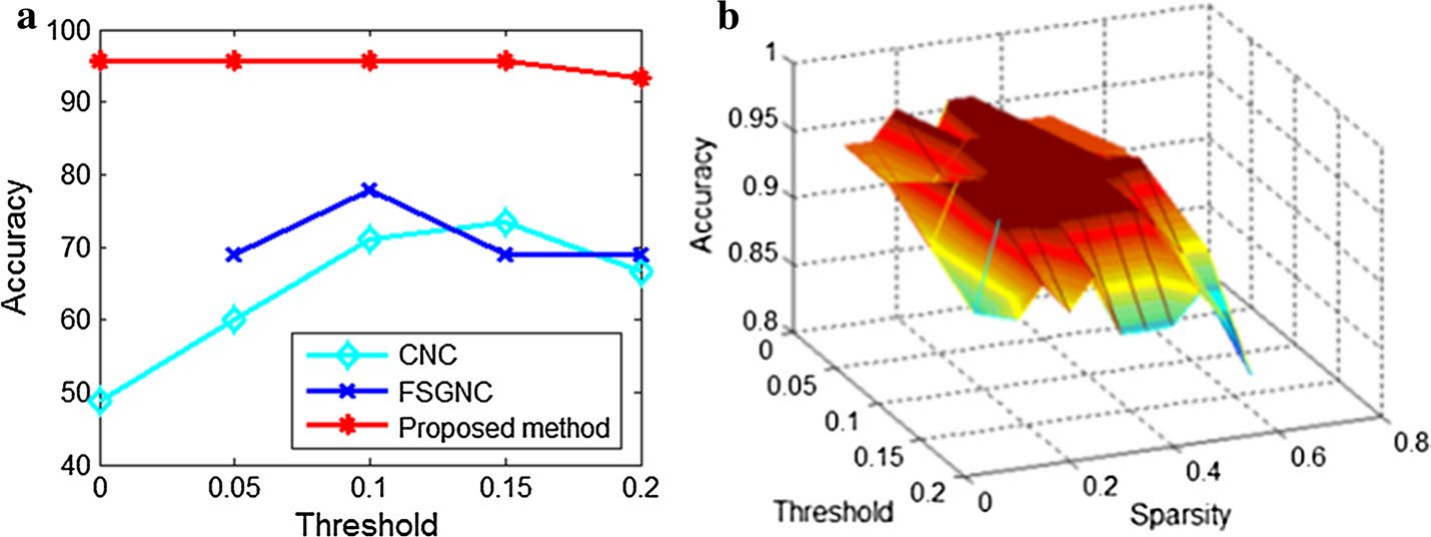
**a** Variations of accuracies of our method, CNC and FSGNC versus network threshold, **b** variations of accuracies of our method versus network threshold and sparsity

The leave-one-out cross validation is carried out to assess classification performance. In experiment, we run our algorithm and the other brain network based algorithms including CNC, MTNC, FSGNC and SVM on our dataset. According to classification results of each brain network based algorithm, we counted the number of $TP$, $TN$, $FP$ and $FN$, respectively. Then we calculated the above 5 measures for each brain network based algorithm, and the results are shown in Table 2. The results demonstrate that our method outperforms CNC, MTNC, FSGNC and SVM. The accuracies of these brain network based methods verses the variations of network threshold are shown in Fig. 3a. It demonstrates our method has higher accuracy than the other methods with the same network threshold. We also show the performance of our method versus the variation of threshold and sparsity in Fig. 3b.

**Table 2:** The performance of our method and the other three brain network based methods including CNC, MTNC, FSGNC and SVM

Method	Accuracy (%)	Sensitivity (%)	Specificity (%)	PPV (%)	NPV (%)
CNC	73.33	79.17	66.67	73.08	73.68
MTNC	82.22	87.50	76.19	80.77	84.21
FSGNC	77.78	*100.00*	52.38	70.59	*100.00*
SVM	75.56	83.33	66.67	74.07	77.78
Proposed method	*95.56*	*100.00*	*90.48*	*92.31*	*100.00*

The highest value of each measure is in italics

Several voxel based methods are also compared with our method. Table 3 shows the comparison between our method and several voxel based methods. In Table 3, the accuracies of the these voxel based methods were the results reported in the relevant references [12, 13, 36, 37].

**Table 3:** The accuracies of our method and several voxel based methods

Method	Data	Accuracy (%)
Chyzhyk et al. [36]	Resting state-fMRI (ALFF)	87.67
Chyzhyk et al. [36]	Resting state-fMRI (fALFF)	82.19
Chyzhyk et al. [36]	Resting state-fMRI (ReHo)	84.19
Chyzhyk et al. [36]	Resting state-fMRI (VMHC)	91.19
Du et al. [37]	Resting state-fMRI	93.00
Cao et al. [13]	fMRI during sensorimotor task	83.11
Juneja et al. [12]	fMRI during AOD task	89.70
Juneja et al. [12]	fMRI during AOD task	92.00
Proposed method	Resting state-fMRI	*95.56*

The highest accuracy is in italics

**Fig. 4:**
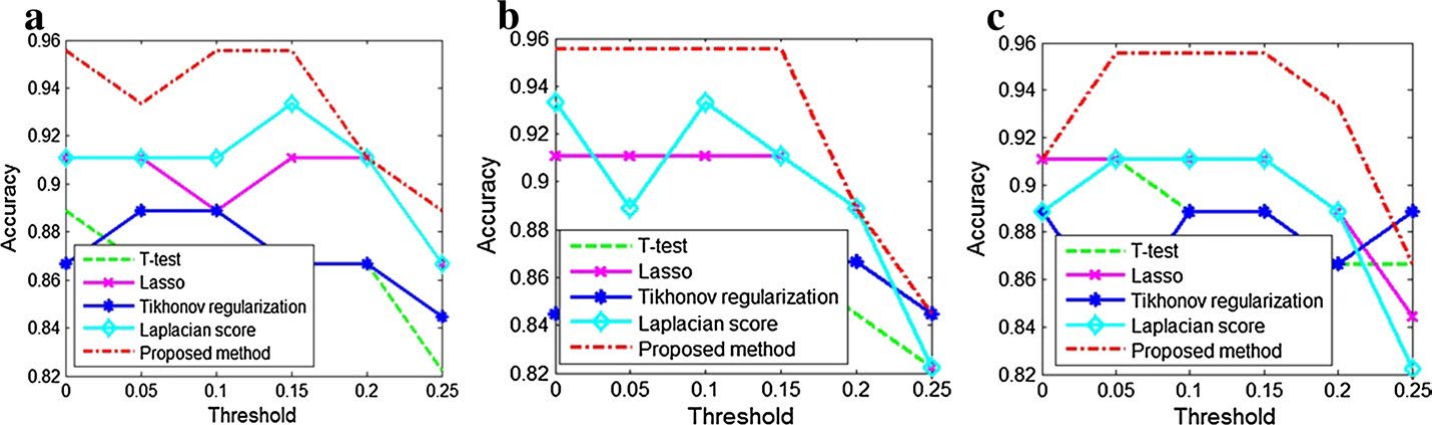
Variations of accuracies of T-test, Lasson, Tikhonov regularization, Laplacian score and our methods versus threshold and sparsity. **a** Sparsity = 0.2, **b** sparsity = 0.25 and **c** sparsity = 0.3

To evaluate the performance of non-negative sparse representation used in our method, several feature selection methods including *t* test, Lasso, Tikhonov regularization, Laplacian and sparsity score are also compared. The accuracies of these methods are shown in Fig. 4.

The functional brain network is a kind of topologically complex network. In experiment, the differences in topological properties of functional network are measured by connectivity strength [22], connectivity diversity [22], clustering coefficient [9], overlap score [35] and weighted overlap score [35]. These topological metrics are calculated in group-level, and the results are shown in Table 5.

### Alternation of the brain connectivity

**Fig. 5:**
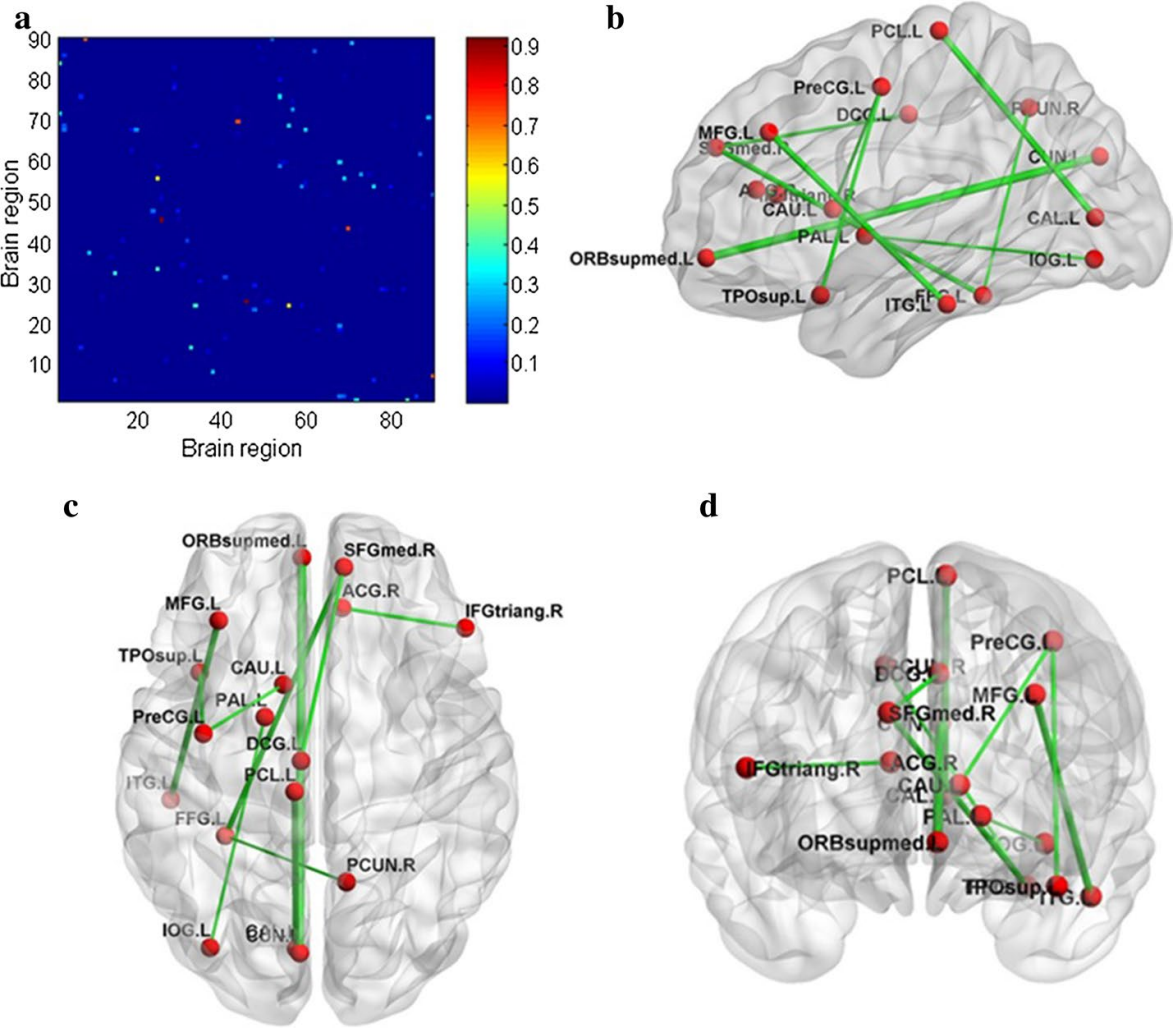
**a** All the discriminative abilities of the connectivities of the brain networks. (The position in ith row and jth column in **a** denotes the connectivity between ith brain region and jth brain region, and its weight can be judged by the corresponding color). **b**–**d** The top 10 alteration connectivities for identifying schizophrenia

We also indicate the biomarks for identifying schizophrenia by extracting its alteration of the brain connectivity. In our method, the discriminative ability of each connectivity for identifying schizophrenia is learned by non-negative elastic-net. We show all the discriminative abilities of the connectivities of the brain network in Fig. 5a. In Fig. 5a, both the horizontal axis and the vertical axis stand for the brain region index used in Automated Anatomical Labeling (AAL) temple [38]. For example, the position in ith row and jth column in Fig. 5a denotes the connectivity between ith brain region and jth brain region, and its weight can be judged by the corresponding color. According to the rank of the discriminative abilities of the brain connectivities, we choose the top 10 significant alteration of connectivities (SAC) and show their positions in brain by Fig. 5b–d. Table 4 gives the top 30 SACs between schizophrenia and healthy control.

**Table 4:** Significant alteration of connectivity (SAC) between schizophrenia and healthy control

No.	Brain area A	Brain area B	Weight score
SAC 1	Cuneus	Superior frontal gyrus, medial orbital	41.36
SAC 2	Paracentral lobule	Calcarine fissure and surrounding cortex	32.57
SAC 3	Inferior temporal gyrus	Middle frontal gyrus	31.04
SAC 4	Fusiform gyrus	Superior frontal gyrus, medial	25.79
SAC 5	Temporal pole: superior temporal gyrus	Precentral gyrus	19.16
SAC 6	Median cingulate and paracingulate gyri	Superior frontal gyrus, medial	17.84
SAC 7	Anterior cingulate and paracingulate gyri	Inferior frontal gyrus, triangular part	17.02
SAC 8	Caudate nucleus	Precentral gyrus	15.79
SAC 9	Lenticular nucleus, pallidum	Inferior occipital gyrus	14.57
SAC 10	Precuneus	Fusiform gyrus	14.45
SAC 11	Precuneus	Superior parietal gyrus	13.76
SAC 12	Hippocampus	Middle frontal gyrus	13.53
SAC 13	Temporal pole: middle temporal gyrus	Paracentral lobule	12.73
SAC 14	Caudate nucleus	Fusiform gyrus	11.26
SAC 15	Precuneus	Supplementary motor area	10.44
SAC 16	Postcentral gyrus	Inferior occipital gyrus	9.99
SAC 17	Inferior temporal gyrus	Middle occipital gyrus	9.57
SAC 18	Lingual gyrus	Superior frontal gyrus, medial	9.45
SAC 19	Precuneus	Precentral gyrus	9.26
SAC 20	Middle temporal gyrus	Inferior parietal, but supramarginal and angular gyri	8.40
SAC 21	Precuneus	Precentral gyrus	8.03
SAC 22	Parahippocampal gyrus	Anterior cingulate and paracingulate gyri	7.46
SAC 23	Middle temporal gyrus	Precentral gyrus	6.99
SAC 24	Inferior frontal gyrus, triangular part	Superior frontal gyrus, orbital part	6.31
SAC 25	Lenticular nucleus, pallidum	Superior frontal gyrus, orbital part	5.97
SAC 26	Lingual gyrus	Insula	5.72
SAC 27	Thalamus	Gyrus rectus	5.40
SAC 28	Precuneus	Rolandic operculum	5.13
SAC 29	Heschl gyrus	Inferior occipital gyrus	4.72
SAC 30	Supramarginal gyrus	Median cingulate and paracingulate gyri	3.84

## Discussion

For the classification performance, our method achieves the accuracy of 95.56% with the sensitivity of 100%. The experimental results imply that schizophrenia patients may have the altered graph structure of many brain region connectivities and our method is more appropriate in extracting the altered pattern than other methods. Figure 3 demonstrates that our method is robust to the variations of network threshold and sparsity.

As a brain network based method, our method achieves higher accuracy than theses voxel based methods shown in Table 3. More importantly, although our method is performed on resting state-fMRI data, which is usually considered more difficult than classification on fMRI during special task, it still achieves higher accuracy than those methods performed on fMRI during sensorimotor task or auditory oddball (AOD) task. In addition, our method is much more efficient than those voxel based methods in computational cost. Because the number of the brain region connectivity used in our method is much smaller than the voxel number or voxel connectivity number used in voxel based methods.

As can be seen from Fig. 4, our method outperforms t-test, Lasso, Tikhonov regularization, Laplacian and sparsity score. It is noteworthy that although fully connected brain network (brain network threshold is set to 0) are directly used for feature selection and classification, our method still achieves better performance. It implies there exist some weak connectivities having discriminative ability for classification in brain network. As can be seen from Fig. 5a, the discriminative abilities of the connectivities are very sparse, which is a good property for determining biomarks in clinical trials.

Among the related brain regions of the top 10 SAC, cuneus, superior frontal gyrus, medial, middle temporal gyrus, superior temporal gyrus, median cingulate and paracingulate gyri, inferior frontal gyrus, triangular part and precuneus belong to default mode Network (DMN) [39, 40], which is a group of areas in the human brain characterized, collectively, by functions of a self-referential nature. Buuren et al. also indicates that the alteration of the brain network of schizophrenia and Alzheimer are closely related to DMN [41]. Furthermore, Precentral_L and Paracentral_Lobule_L related to the movement are also selected by our method. Recent studies show that these movement related regions are very important for identifying schizophrenia patients with psychedelic covet condition [42]. As we know, there are positive and negative values produced by the previous feature extraction method, such as Lasso. Because of introducing the non-negative constraint term, all the weight score of the brain connectivity by using the proposed method are positive values, which enable us to rank all the brain connectivities with a uniform criterion.

The results shown in Table 5 demonstrate that the difference between schizophrenia and healthy control in connectivity strength is generally small and not significant. The connectivity diversity of schizophrenia patients group is higher than that of healthy controls group. In other words, there is a variety of differences in brain connectivities between patients, which might be viewed as the functional network evidence for why schizophrenia is a highly heterogeneous disease. The clustering coefficient of the functional network of schizophrenia patients group is lower than that of healthy controls group. It demonstrates that a part of the connectivities may diminish or completely disappear in the functional network of patient. The overlap score and weighted overlap score are designed to capture the influence of network difference from each group [35]. Compared to the other three topological metrics, both of the overlap score and weighted overlap show more obvious difference between the schizophrenia patients and healthy controls. These two metrics are proper in distinguishing schizophrenias and healthy controls in group-level.

**Table 5:** Functional connectivity and network topological metrics for healthy controls and schizophrenia patients

Topology metrics	Healthy controls (mean ± SD)	Schizophrenia patients (mean ± SD)
Connectivity strength	6.3853 ± 2.3951	6.1172 ± 2.5732
Connectivity diversity	0.0138 ± 0.0087	0.0152 ± 0.0095
Clustering coefficient	1.1135 ± 0.0937	1.0690 ± 0.1059
Overlap score	0.0538 ± 0.0169	0.0461 ± 0.0042
Weighted overlap score	0.0118 ± 0.0060	0.0091 ± 0.0014

## Conclusion

In this paper, we proposed a simple and effective feature selection method for identifying schizophrenia. On a real dataset, it achieved the sensitivity, specificity and accuracy of 100, 90.48 and 95.56%, respectively. Compared to conventional brain network significant feature extraction methods, the proposed method can simultaneously alleviate the small sample problem and rank the significance of the connectivity with a uniform criterion. The proposed method not only obtained good performance in classification accuracy, but also provided biomarkers to guide the schizophrenia diagnosis.
